# Experimental prosopis management practices and grassland restoration in three Eastern African countries

**DOI:** 10.1186/s43170-023-00163-5

**Published:** 2023-07-13

**Authors:** René Eschen, Ketema Bekele, Yohana Jumanne, Staline Kibet, Fernadis Makale, John Richard Mbwambo, Berhanu Megersa, Mahamood Mijay, Francis Moyo, Linus Munishi, Mickfanaka Mwihomeke, Winnie Nunda, Moses Nyangito, Arne Witt, Urs Schaffner

**Affiliations:** 1 CABI, Delémont, Switzerland; 2 Haramaya University, Dire Dawa, Ethiopia; 3 The Nelson Mandela African Institution of Science and Technology, Arusha, Tanzania; 4 Tanzanian Forestry Research Institute, Lushoto, Tanzania; 5 Land Resource Management and Agricultural Technology (LARMAT), University of Nairobi, Nairobi, Kenya; 6 CABI, Nairobi, Kenya; 7 Werer Agricultural Research Center, Afar Region, Ethiopia; 8 Tanzanian Forestry Research Institute, Moshi, Tanzania

**Keywords:** Invasive alien plant management, *Prosopis juliflora*, East Africa, Woody invasive species, Grassland restoration

## Abstract

Woody species have been introduced in many parts of the world to provide economic benefits, but some of those species are now among the worst invaders, causing widespread economic and environmental damage. Management of woody species to restore original ecosystem services, such as biodiverse grassland that can provide fodder and sequester carbon, are needed to limit the impacts of alien species. However, the best management methods, i.e., the most economically efficient and effective way to remove trees and the most effective way to restore or rehabilitate the cleared land, are not developed for many species. In Eastern Africa, prosopis (*Prosopis julifora*) has invaded large areas of savanna and grassland, thereby affecting, among other things, fodder and water for livestock, access to dry season grazing lands and ultimately pastoral livelihoods. We tested three prosopis treatments (manual uprooting and cut stump and basal bark herbicide application) in combination with three incremental restoration interventions (divots, divots + mulching, divots + mulching + grass seed sowing). The three-year study was replicated in Ethiopia (Afar National Regional State), Kenya (Baringo county) and Tanzania (Moshi district). Prosopis survival and vegetation development, both diversity and biomass, were recorded. The prosopis treatments were all highly effective (between 85 and 100% tree mortality in almost all cases), but the two treatments that involved the complete removal of the aboveground biomass (manual and cut stump) yielded a more productive and more diverse vegetation than the treatment that killed the trees standing (basal bark). Compared to the effect of prosopis removal, the effect of restoration interventions on vegetation composition was small, indicating that most species re-established from the soil seed bank. The results show that it is possible to restore land previously invaded by prosopis. Despite the different rates of vegetation establishment and variation in species composition, the restoration interventions resulted in vegetation that in some cases contained a substantial fraction of perennial grasses. The method chosen to control prosopis depends on the availability of resources, including herbicides, and the need to remove rootstocks if the intention is to plant crops.

**Supplementary Information:**

The online version contains supplementary material available at 10.1186/s43170-023-00163-5.

## Introduction

Global change drivers, including Invasive Alien Species (IAS), have serious negative impacts on the environment and livelihoods (Linders et al. 2020; Sala et al. 2000; Shackleton et al. 2019). Woody alien species have been introduced to many parts of the world to provide economic benefits, but some of those species are among the worst invaders known, causing widespread economic and environmental damage. While IAS can provide benefits to some members of society, the environmental changes caused by them affect the livelihoods of the majority of people, especially in regions where the dominant livelihood is (agro-) pastoralism. The more these IAS spread and displace native vegetation, the more they negatively affect the natural resources that many people depend on, as well as traditional sources of income and cultural goods (Bekele et al. 2018). There is thus an urgent need for management of such IAS and for subsequent restoration or rehabilitation of the cleared land to safeguard traditional livelihoods and ecosystem services. Such interventions may not only reduce impacts on people’s current livelihoods by increasing fodder and water availability to livestock, but may also provide environmental benefits such as reduced erosion or enhanced soil carbon sequestration (Eschen et al. 2021a, b). People in affected areas are aware of the negative effects of IAS and are willing to invest in management of the IAS (Bekele et al. 2018), but there is a lack of tested options to achieve this reversal of impacts, which hampers informed decision making by communities whose land has been invaded.

When management of woody IAS is implemented in a sustainable way, the trees must be killed and, if the land is subsequently used for agriculture, the rootstocks removed. Killing of woody IAS can be achieved through chemical and physical methods (Nuñez et al. 2017; Sádlo et al. 2017). While management using machines such as bulldozing and chain-pulling may be applicable for large areas, manual and chemical methods may be especially suitable for treatment of small and localized invasions, especially where resources are scarce. For example, local eradication of an IAS at the front of an invasion to slow down or stop its further spread often targets individual young trees (Brundu et al. 2020), which may be uprooted by pulling or digging, and small stands of trees too large to be uprooted may be treated by targeted spraying of herbicide on the lower parts of the trunk (basal bark treatment) or on the stump immediately after the trunk has been cut (cut stump treatment). There are differences in implementation cost and practical applicability of particular interventions, resulting in context-dependent preferences: leaving dead aboveground biomass in place may provide shading for sown species, whereas removal of aboveground or belowground biomass would allow access to the land for cattle or farming activities. Comparative knowledge about the cost and effectiveness of control, and especially restoration methods is scarce, yet it would be useful to inform future management of woody IAS.

Restoration of grassland ecosystems is the acceleration of vegetation development through practices that promote the establishment of desired species (Gann et al. 2019), such as native perennial bunch grasses that are good fodder species, particularly in semi-arid and arid grasslands. The rapid establishment of perennial vegetation would provide ecological and livelihood benefits, such as carbon sequestration, fodder and protection against erosion Mbaabu et al. 2020) and reduce the establishment of weeds.

Acceleration of vegetation development may be necessary if sufficient propagules are present but the conditions for establishment are unfavourable, or if propagules of desired species are not present. Creation of small topographic structures, such as ridges or divots, may collect rain water and seeds may enhance establishment of species that are present in the seed bank through the creation of safe sites for seedling emergence (Visser et al. 2007; Wagner et al. 2016). Hay spread on the surface of the soil may act as mulch to create a shaded environment that helps in maintaining soil moisture, reducing soil moisture loss and suppressing weeds (Valkó et al. 2022), as well as moderating soil temperatures (Baasch et al. 2016; Shaw et al. 2020), which can promote seedling emergence and establishment. Finally, sowing of desired species, either by adding seeds or serve as a source of grass seeds of by spreading seed-rich hay, can reintroduce or increase the density of species of which viable seeds are no longer present in the soil seed bank (Shaw et al. 2020), but more research is needed to support species specific restoration measures. The selection of a certain plant introduction measure should fit the local conditions and the available resources.

On restored pastures, a grazing management is needed that reduces the risks of overgrazing and allows permanent establishment and regrowth of fodder species (Mureithi et al. 2014; Wairore et al. 2015). Such grazing management may include rotational grazing and temporary banning access to grazing areas to allow recovery of the vegetation, which are common traditional practices in Eastern African (agro-) pastoralist communities (Mureithi et al. 2014; Wairore et al. 2015).

Invasion of Arid and Semi-Arid Lands (ASALs) by woody IAS such as *Prosopis juliflora* (Sw.) DC. (Fabaceae; prosopis hereafter) are associated with reductions in biodiversity, water availability and grazing capacity for both wildlife and livestock. Controlling plant invasions and restoring degraded areas are therefore important undertakings to ensure sustainability of these ecosystem services. Prosopis is a highly invasive, spiny shrub or tree that is native to Central and northern South America (Kaur et al. 2012), which was introduced to Eastern Africa in the 1970 s as a wind break in degraded habitats and to provide a source of fodder, fuel wood and timber (Mwangi and Swallow 2008). Today, prosopis is known to spread rapidly and transform socio-ecological systems: the tree negatively impacts livelihoods in many ways, including through deterioration of grassland and livestock-based income in pastoralist-dominated regions (Bekele et al. 2018; Eschen et al. 2021a, b; Linders et al. 2020), reductions in biodiversity and water (Dzikiti et al. 2013; Linders et al. 2019; Shiferaw et al. 2021), and increased densities of malaria-transmitting mosquitoes (Muller et al. 2017). Removal of prosopis from invaded land would mitigate some of its negative impacts, while restoration of grassland would strengthen pastoralist livelihoods and finally contribute to climate change mitigation (Eschen et al. 2021a, b; Mbaabu et al. 2020).

Prosopis management has been undertaken in many countries where the species has been introduced and it has become invasive, with different approaches taken, depending on the country, resource availability and the extent of the invasion. Consequently, there have been varying degrees of success. For example, in Australia management relies on a combination of physical removal using bulldozers, chemical treatment of small patches and biological control using seed feeding bruchid beetles (genus *Algarobius*) and a leaf tying moth (genus *Evippe*) (Osmond et al. 2003). Especially biological control by the leaf tying moth can significantly reduce seed production and even kill trees that are drought stressed (Van Klinken and Campbell 2009). In Eastern Africa, management through utilisation has been promoted in particular by the Kenyan government, and can be described as harvesting of prosopis aboveground biomass for making charcoal or use of the pods as animal feed in an effort to reduce the trees’ abundance and spread. Similarly, several international NGOs promote utilisation for charcoal making as a way to manage prosopis in Eastern Africa, while also aiming to support livelihoods. However, there is no evidence that utilisation has resulted in lasting reductions in abundance or spread (Mbaabu et al. 2019). One reason for the failure to control prosopis in these efforts is that removing aboveground biomass for making charcoal does not kill the trees, which coppice profusely and produce flowers and seeds within few months. Thus, the most successful and cost-effective way of permanent removal of prosopis is not known.

We tested different prosopis management methods in combination with grassland restoration interventions in three Eastern African countries in order to identify methods that are effective and applicable across the region and to compare the time required for mechanical and chemical control methods.

We tested the following hypotheses: (1) all three prosopis management methods are equally effective, but basal bark takes the least and manual removal the most time to implement; (2) Management of prosopis without removal of above-ground biomass (basal bark treatment) results in the more rapid establishment of herbaceous species than interventions that remove the aboveground biomass (manual uprooting and cut stump treatments); (3) Combining prosopis management with restoration interventions accelerates vegetation establishment and leads to higher vegetation cover and species number; (4) Sowing grasses promotes establishment of desired indigenous grass species (through mulching and sowing), as opposed to divots that promote establishment of all species.

## Methods

### Study areas

The study was conducted in three countries in order to assess the applicability of the tested methods in different regions that represent the geographic extent and some of the habitat types that are affected by prosopis in eastern Africa. The climate of the three sites was roughly comparable (arid and warm), but the history of invasion, land use and disturbance varied across the sites, thus potentially allowing somewhat more general conclusions about the results. It was impossible to have multiple sites per country as a result of budgetary restrictions. However, testing of chemical control options in three countries is highly relevant, as all three countries have recently adopted national strategies for dealing with invasive species or prosopis specifically, yet chemical means of controlling the species remain unavailable in these countries, because no herbicides have been approved or tested for use against prosopis. Herbicides for use against prosopis have been tested and registered for use in South Africa and Australia, where herbicide applications are part of prosopis management. The results of this study were therefore expected to support decision making concerning prosopis management.

### Ethiopia

The site in Ethiopia was located in Afar National Regional State (39°34′–42°28′E, 8°49′–14°30′N). The altitude ranges from 94 m below sea level to 2235 m a.s.l. The region is mainly arid and semi-arid dryland with mean temperatures of 41 ℃ in June and ca. 21 ℃ between November and December (Shiferaw et al. 2019). Rainfall is erratic and scarce (ca. 560 mm per year). Almost 90% of the population are pastoralists. Awash River floodplains, which are the main dry season source of grazing, are invaded or under risk of invasion by prosopis (Linders et al. 2019). The region’s natural vegetation cover consists of patches of scattered dry shrubs, acacia woodland, bushland, grassland and wooded grassland (Engda 2009). However, currently, prosopis is replacing the original vegetation and other land use types (grassland, bare land, bush-shrub-woodland and natural forests). The invasion causes biodiversity loss, reduced water availability and mobility loss and ultimately resulted in significant negative impact of local livelihoods, mainly pastoralism (Bekele et al. 2018).

### Kenya

In Kenya, the study was conducted in the Njemps flats of Baringo County (35°57′–36°12′E, 0°02′–0°44′N, ca. 1000 m.a.s.l.). The area experiences hot and dry conditions throughout most of the year with maximum and minimum temperatures of 30–35 and 16–18 ℃. Rainfall is highly variable, both annually and interannually. Average annual rainfall is 650 mm with weak bimodal peaks recorded from March–May and June–August. High evapotranspiration and low, variable rainfall create water scarcities that limit intensive agricultural use (Andersson, 2005). The inhabitants are therefore largely (agro-) pastoralists keeping cattle, sheep and goats and engaging in small-scale cultivation. *Prosopis juliflora* was first introduced in the area on an extensive scale in 1983 through the Fuelwood Afforestation Extension Project (Getahum 1988; Kariuki 1993). The project aimed at involving local communities in tree planting for mitigating problems such as lack of firewood and decreasing vegetation cover (Kariuki 1993). In total, more than 20 plantations were established, covering an area of over 250 ha. Historically, the area was dominated by classic savanna vegetation such as Acacia trees (mainly *Vachellia tortilis)* in association with *Boscia* spp and *Balanites aegyptiaca* and bushes of *Salvadora persica*. Generally, the understory lacked permanent herbaceous cover but ephemeral herbs appeared during wet season. The prosopis invasion now covers an area of 1,276 km^2^ (Mbaabu et al. 2019), with significant impacts on water resources, use and livelihoods.

### Tanzania

In Tanzania, the study was conducted in Kahe Ward in Moshi District (ca. 37°25′E, 3°29′ S). The area lies in the semi-arid lowlands between 450 and 680 m above sea level and average annual rainfall of 365 mm, with most rain occurring between March and May (de Bont et al. 2019). Temperature ranges from 14 ℃ to 35 ℃, with January being the hottest month. The main economic activities are crop cultivation (maize, beans and tomatoes) which rely mostly on irrigation from existing canals, rivers and wells, and livestock keeping dominated by cattle, sheep and goats. The natural vegetation is characterized by scattered Baobab *(Adansonia digitata), Faidherbia albida* (apple-ring acacia), *Vachellia xanthophloea* (Yellow-bark acacia) and the two shrubs *Suaeda monoica* and *Tamarix nilotica* in areas adjacent to river courses. Prosopis has replaced most of the natural vegetation and it is now the dominant tree species in the area. The species arrived in the area mainly through livestock movements and floods which carry substantial amount of seeds from invaded areas in the north eastern side bordering Kenya (Kilawe et al. 2017). Due to its high densities, prosopis is already posing management challenges by invading agricultural fields under fallow, making crop production laborious and more expensive. It has also replaced grasslands and thereby reducing the land available for livestock grazing.

### Experimental design

**Fig. 1:**
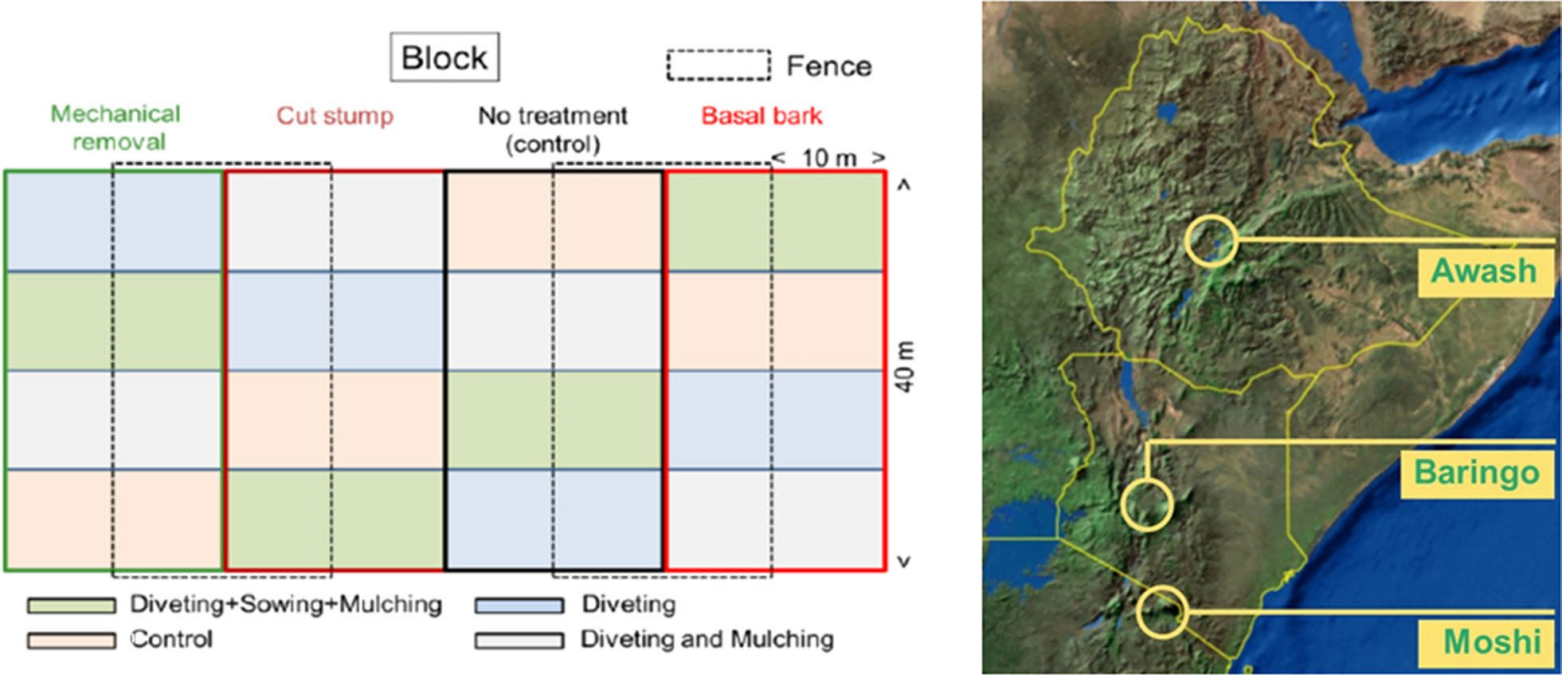
Schematic representation of treatment allocation in a block (left) and map with approximate locations of the study regions in Eastern Africa (right)

The field sites were located on habitats invaded by prosopis, with initial prosopis cover > 80% with similar densities within each site. In each country, three blocks of 40 × 80 m each were established at a single site in late 2016 or the first half of 2017. Thus, there were nine blocks. The blocks in each country were spatially separated because large, homogeneous areas with high prosopis cover were not available. The distance between the blocks varied from ca. 100 m in Tanzania to ca. five km in Kenya. Each experimental block comprised four plots (40 × 20 m) that were randomly assigned to one of four prosopis treatments (Fig. 1). The entire blocks were “fenced” with cut prosopis branches to protect the experimental plots from disturbance by livestock. Half of the plots in each block were fenced using barbed wire (10 × 40 m; grazing treatment) and the area within each of the fenced and unfenced areas was divided into four 10 × 10 m subplots that were randomly assigned to one of four restoration treatments.

### Experimental treatments

Three prosopis treatments were tested: Manual removal (MR), cut stump (CS) and basal bark (BB). In addition, there was an untreated control (CO) in each block. In Kenya and Ethiopia, the herbicides used for the field trial were received from the manufacturing company (Arysta LifeScience, South Africa; Table 1) through an authorized import permit for experimental purposes. The active ingredient in the herbicides, which were applied in the cut stump and basal bark treatments, was Triclopyr that interrupts cell division and growth in meristem tissues. In the cut stump treatment, stems were cut with a chainsaw as close to the ground as possible and the herbicide applied to the stump within one minute of cutting the stems using a paintbrush. The basal bark treatment consisted of application of Triclon to the bark of the basal 75 cm of the stem using a knapsack sprayer or a paintbrush (in Tanzania only). The manual treatment consisted of the removal of aboveground biomass and uprooting of the rootstock to a depth of ca. 30 cm using picks and hoes.

**Table 1:** Description of the chemical control treatments applied in the field trials in Kenya, Tanzania and Ethiopia

Country	County/region	Basal bark treatment	Cut stump treatment
Kenya	Baringo	Triclon (480 g/l Triclopyr as butoxyl ethyl ester), mixed with diesel (2L Triclon/100L diesel); applied with knapsack sprayer	Kaput 100 gel (50 g/kg Picloram as potassium salt and 50 g/kg triclopyr as triethylamine salt)
Tanzania	Kilimanjaro	Triclon (undiluted), repeated once on regreening trees (mixed with diesel (10 L Triclon/100 L diesel); applied with low pressure hand sprayer, combined with a brush application on small trees	Triclon (undiluted), repeated once on resprouting rootstocks
Ethiopia	Afar	Triclon, same application as in Kenya	Kaput 100 gel

Half of the prosopis removal plots were fenced to exclude livestock during controlled grazing of the unfenced subplots. However, the fencing did not entirely prevent livestock from entering the fenced plots, sometimes due to livestock keepers looking for scarce grass for their animals. Hence, although we included the fencing treatment in the analysis, we cannot be sure that any differences are due to differences in grazing pressure as we intended.

Three restoration interventions were tested in an additive design: divots (DIV); divots and mulching (DIM) and divots, sowing and mulching (DSM). There was an unaided restoration as control (UNR) plot for every set of restoration interventions. Divots were shallow hollows of 50 × 50 cm size and 10–15 cm deep; 36 divots were made per 10 × 10 m subplot, with the edge of the last divots in every row at least 1 m from the side of the subplot and the centre of each divot was at least 1.5 m from the centre of the neighbouring divot. Divots or other topographic features alone can act to collect rainwater and seeds, which can facilitate vegetation establishment (Holden and Miller 1996; Mousavi et al. 2019). Mulching was done using grass/hay bales which were sourced from a nearby area. Mulch was placed evenly across the subplots, including over the divots, in a layer of 3–5 cm deep. The mulch was dry when applied, which may have resulted in lower numbers of seeds than when freshly cut grass would have been applied. Thus, the effect of mulching was likely primarily shading. A seed mixture consisting of native grasses was sown evenly in the relevant subplots at a seeding density of 2 g/m^2^. In Kenya the seeds were obtained from the Rehabilitation of Arid Environments Charitable Trust (RAE Trust) which harvests seeds from grass stands in and around Baringo county and who are involved in degraded land reclamation through planting and production of indigenous grass and tree species. In Ethiopia, grass seeds were obtained from the Worrer Station of the Ethiopian Institute of Agricultural Research, where seeds of various grass species are produced. In Tanzania the seeds for the experiment were harvested by hand from grasslands in the vicinity of the experimental site. In all countries, the main grass species was *Cenchrus ciliaris* L., a productive and highly palatable species commonly sown on grassland restoration sites in Eastern Africa.

### Measurements

In Kenya and Tanzania, the stem base diameter (SBD; 30 cm above ground) of all prosopis stems (including multiple stems from the same rootstock) was recorded in each plot prior to the implementation of the treatments (Additional file 1). In Ethiopia, SBD was not measured. The time needed for implementation of the prosopis treatment was recorded for each plot. There was variation in the time needed depending on, among other factors, the time of day, the person implementing and prosopis density. However, treatments were applied over multiple days and by groups of people and consequently we don’t expect treatment bias.

The survival of trees in the three trials was recorded at different time intervals, as either the lack of regrowth from cut stumps or general drying of leaves on the trees that were treated with basal bark herbicide application. In Kenya, survival was recorded 33 months after treatment application to allow for adequate time for herbicide action to be completed and to observe for any coppicing or tree regeneration to occur. In Ethiopia, survival of trees was recorded three, 6 and 9 months after treatment application. In Tanzania, survival was assessed after 7 months; trees that were not killed by the initial chemical treatments were treated again and survival was recorded for a second time 19 months after the initial treatment application. Prosopis seedlings were removed in all plots in the three countries; the number of seedlings was counted in Kenya every time vegetation assessments were made and in Tanzania the number of removed seedlings was counted 7, 8, 15 and 20 months after the start of the experiment.

Species richness and cover of herbaceous vegetation in three randomly selected 1 m^2^ plots within the central 5 × 5 m area of quadrat was assessed by counting the number of cells of a point frame where each species was rooted (Floyd and Anderson 1987). Species richness and cover did not include prosopis. Identification of species was done with the help of a local botanist and samples of unidentified species were collected for identification. A few species at the Kenyan site could not be identified to species or genus level. None of these species were abundant and in absence of a species name these were coded within each sampling occasion. The presence of additional species in the whole 5 × 5 m square was assessed by randomly walking around for 10 min.

Grass biomass was quantified using a pasture meter (Bransby and Tainton 1977; Douglas and Crawford 1994) in three randomly selected locations within the central 5 × 5 m area of each subplot of the Tanzanian site. The pasture meter disc was 45 cm in diameter and weighed 1500 g. The pasture meter was calibrated for each country to account for regional differences in species composition. For the calibration, a pasture meter reading was taken and the vegetation underneath the disc clipped and oven dried for 24 h at 60 ℃ before assessing dry weight.

### Statistical analysis

Analysis of the univariate parameters was done using generalised linear mixed effects models using the glmmTMB function from the glmmtmb package (Brooks et al. 2017). The time for implementation and effectiveness (mortality) of prosopis treatment; total vegetation cover and species number of the vegetation were used as response variables, and prosopis treatment, grazing and restoration interventions as explanatory variables. The effect of the prosopis management treatments was tested against the prosopis treatment x block interaction, the effect of grazing against the prosopis treatment x block x grazing interaction and the effect of restoration interventions was tested against residuals. Differences among treatments and interventions was assessed using pairwise tests using the emmeans function from the emmeans package P values were adjusted for multiple comparisons using the Tukey method.

There were large differences in the collected variables among the three countries. In Ethiopia only implementation time and mortality were assessed, but data were collected for fenced and unfenced subplots together. The data concerning implementation time and tree mortality collected from the Ethiopian plots were therefore divided by two in order to have the same base unit size (100 m^2^) for all analyses. In Kenya, mean SBD was only in two prosopis treatments and a single of the three blocks and we therefore analysed the effect of prosopis treatments and restoration interventions using a generalised linear model, using the fenced and unfenced subplots as replicates. Vegetation biomass, assessed as dry weight and using a pasture meter, was measured once and only in Tanzania.

Data from the three countries were analysed separately and all statistical analyses were carried out using R (R Core Team 2020). We assessed treatment effects on the evolution of vegetation composition, i.e. multivariate data, with principle response curves (PRCs; (Van den Brink and Braak 1999) using the vegan package (Oksanen et al. 2020). For Kenya and Tanzania, we produced PRCs for the entire study duration and for each sampling event (or selected events), the latter to disentangle importance of prosopis treatment vs restoration intervention. We omitted species that were recorded in fewer than 10 subplots from the multivariate analyses of vegetation composition to avoid outsize influence of uncommon species on the results and we log-transformed the abundance data prior to analysis. We assessed which plant species were significantly associated with particular treatments with indicator species analyses, implemented in the function Indval of the package indicspecies (Caceres and Legendre 2009).

## Results

### Prosopis treatment

**Fig. 2:**
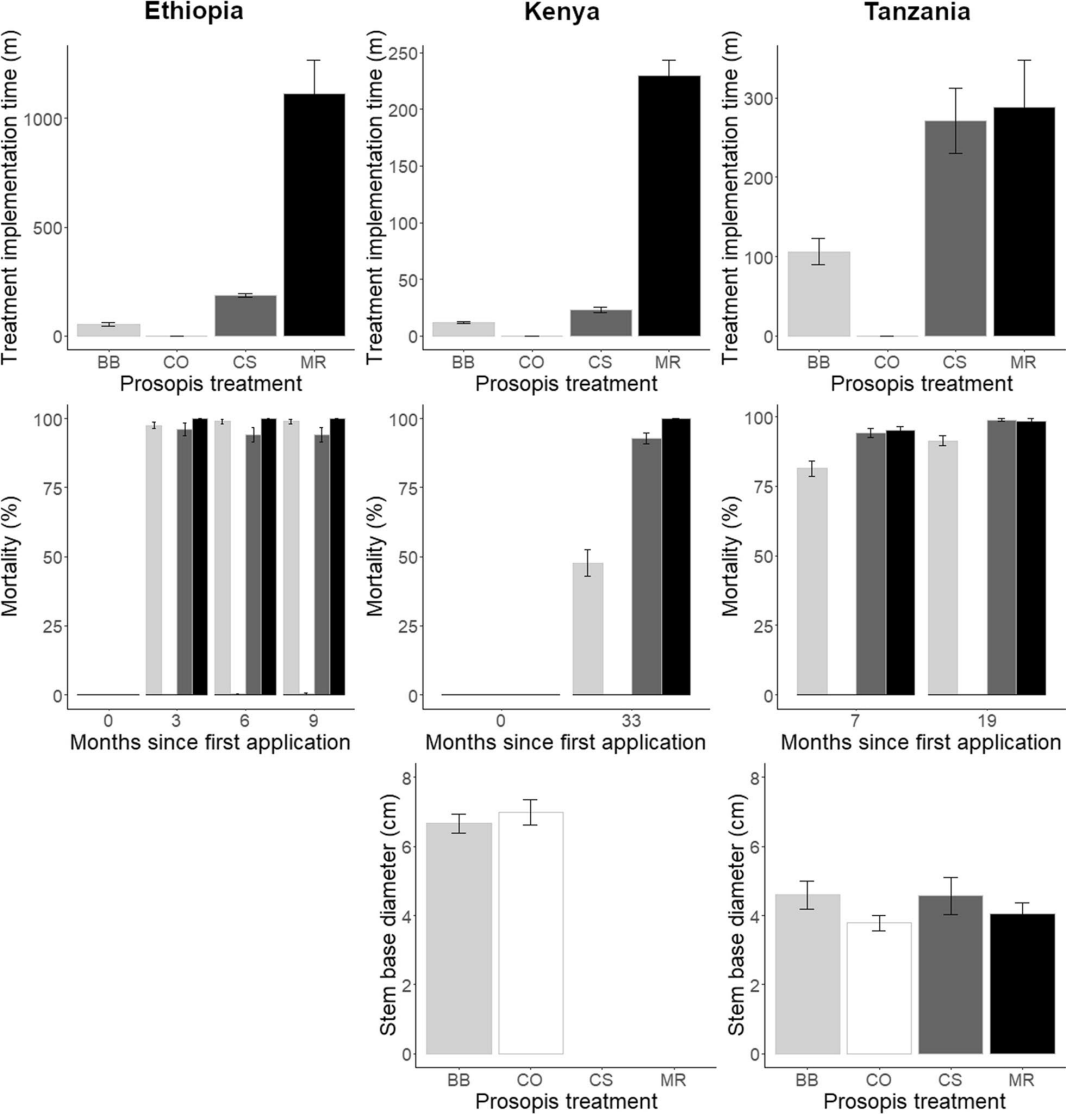
Prosopis treatment implementation time, effectiveness (mortality) of prosopis treatments and stem base diameter in the three countries (from left to right: Ethiopia, Kenya and Tanzania). BB, CS, MR and CO indicate the basal bark, cut stump, manual removal and untreated control treatments, respectively, which correspond to light grey, dark grey, black and white bars. Stem base diameter was measured prior to treatment application. Error bars indicate SE

Treatment implementation time was highest in the manual removal treatment and lowest for the basal bark treatment (Fig. 2). Time to implement cut stump was intermediate, which may have been the result of the need to remove the cut trees from the plots. Overall, the prosopis treatments were all effective at killing prosopis trees, with consistently very high mortality rates in the cut stump and manual removal treatments (Fig. 2). Effectiveness of basal bark was lower in Kenya and Tanzania, possibly due to larger stem diameter or ease of access for application of the herbicide (see below).

In Kenya, the time spent on implementation of the treatments was longest for the manual removal and shortest for the basal bark treatment (P < 0.001; Additional file 2). Time spent on cut stump was intermediate (and significantly different from either of the other two treatments). Manual removal took roughly 20 and ten times longer than basal bark and cut stump, respectively. There was a significant difference among the restoration treatments and an interaction in treatment implementation time between the removal and grazing treatments (P < 0.001), which is likely due to differences in the number of trees and stems in the individual plots. No time was spent on removing prosopis in the control treatment. Mortality in the basal bark was only half of that in the cut stump treatment 33 months after implementation of the treatments (44.9 vs 92.6%, P < 0.001; Additional file 2; Fig. 2). There were no differences in mean SBD among the prosopis treatments, but the significant interaction with restoration interventions reflects the variability among plots. No systematic variability was found. The mean SBD was 6.3 cm (± 0.23).

In Tanzania, mean implementation time for the prosopis treatments was shortest in basal bark and longest in manual removal plots (106 and 288 person hours per plot, respectively; P = 0.002; Additional file 2). Cut stump was intermediate and not different from either of the other two treatments. Significant interacting effects of the prosopis treatments and restoration interventions, and of the prosopis treatments and fencing on mean implementation time were found, but no clear patterns were identified (both P < 0.001). There were also significant differences among the restoration interventions, but no clear pattern was found. Mortality increased over time, from 90.1 to 96.2% seven and 19 months after the start of the study, respectively (P < 0.001; Additional file 2). Moreover, mortality was lower in basal bark than in the cut stump and manual removal treatments (86.3, 96.5 and 96.9%, respectively; P < 0.001). There were no differences in mean SBD among the prosopis treatments or among restoration interventions (P > 0.05; Additional file 2). The mean SBD was 4.25 cm (± 0.19).

**Fig. 3:**
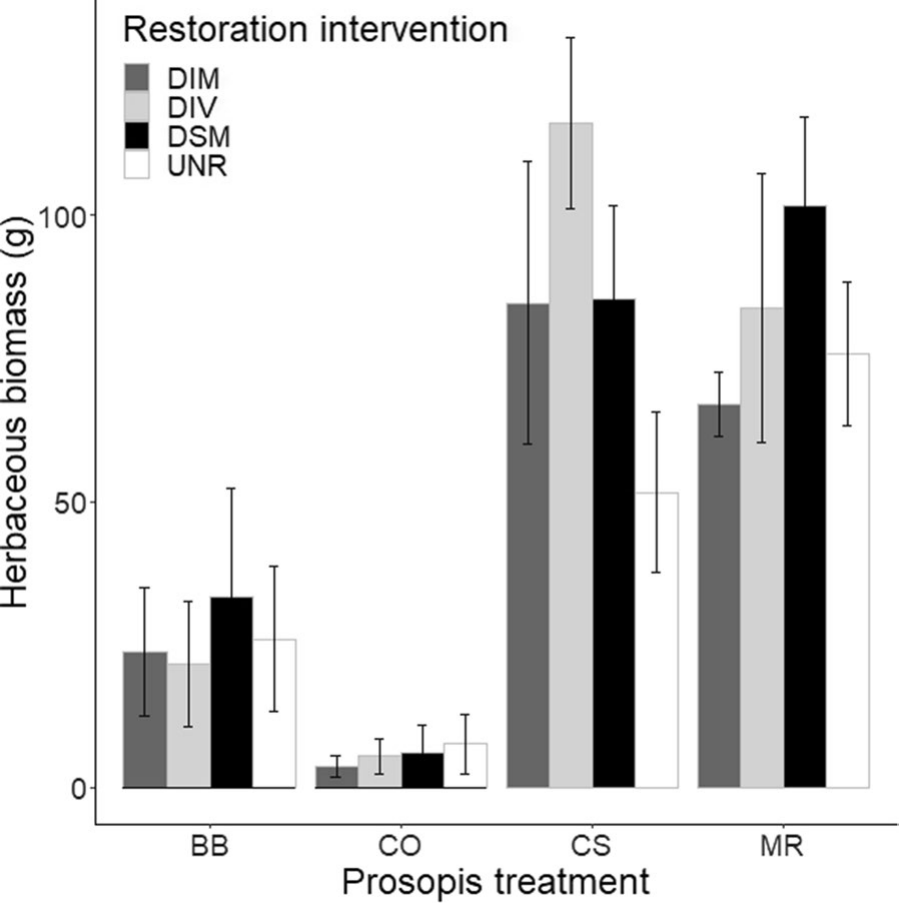
Mean herbaceous vegetation biomass in prosopis treatments and restoration interventions at the Tanzanian site. BB, CS, MR and CO indicate the basal bark, cut stump, manual removal and untreated control treatments, respectively. The shades of the bars indicate restoration interventions and the abbreviations DIV, DIM, DSM and UNR stand for divoting, divoting and mulching, divoting, mulching and sowing, and the unaided restoration treatments. Error bars indicate SE

In Ethiopia, the time spent on implementation of the removal of prosopis was longest for the manual removal and shortest for the basal bark treatment (P < 0.001; Additional file 2). Time spent on cut stump was intermediate and significantly different from either of the two other treatments (P < 0.05). Manual removal took roughly 20 and five times longer than basal bark and cut stump, respectively. There was a significant difference among the restoration interventions and an interaction in treatment implementation time between the prosopis treatments and restoration interventions (P < 0.001), which is likely due to differences in the number of trees and stems in the individual plots. There were no differences in mortality among the basal bark, cut stump and manual removal treatments (P = 0.068; Additional file 2). The average mortality was 97.7%, with manual removal reaching 100% mortality). There were no differences in mortality among the months since start of the study (Fig. 3).

### Prosopis seedling emergence following prosopis management

**Fig. 4:**
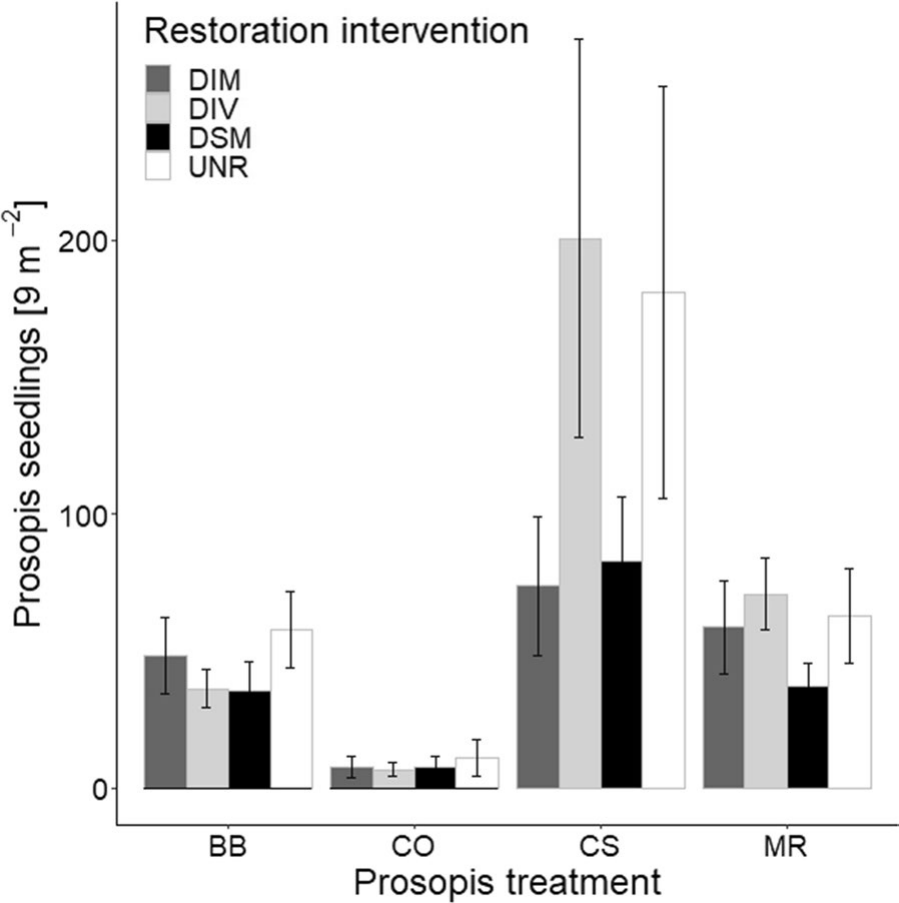
Mean number of prosopis seedlings in prosopis treatments at the Tanzanian site, eight months after the start of the experiment. BB, CS, MR and CO indicate the basal bark, cut stump, manual removal and untreated control treatments, respectively. The shades of the bars indicate restoration interventions and the abbreviations DIV, DIM, DSM and UNR stand for divoting, divoting and mulching, divoting, mulching and sowing, and the unaided restoration treatments. Error bars indicate SE

In Kenya and Tanzania, the number of prosopis seedlings emerging in the plots following the start of the experiment was highly variable across treatments, but was highest at the first assessment following treatment implementation and then declined over time, except in the control plots. The number of prosopis seedlings in Kenya was largest in plots where prosopis had not been managed (control plots) where divots and mulching were applied as restoration interventions (CO DIV). On average, the number of seedlings in CO plots was three times higher than in the other plots (P < 0.001; Fig. 4). In the CO plots the number of seedlings remained about double the number of that in the other prosopis treatment plots. The number of prosopis seedlings in Tanzania was more than five times higher in CS UNR and CS DIV than in the other treatment combinations when they were counted five months after the start of the study (P < 0.001; Fig. 4). The number of seedlings declined throughout the duration of the study in the majority of the other treatments. Later in the study the number of seedlings per plot was comparatively low (months since start: P < 0.001, average 12.8 and 18.4 seedlings per 100 m^2^, respectively).

### Vegetation biomass

In Tanzania, mean vegetation biomass was strongly affected by the prosopis treatments (P < 0.001; Additional file 2; Fig. 3), with biomass in control treatment the lowest and in cut stump the highest (1.68 ± 0.35 and 19.14 ± 2.76 cm, respectively). Although lower than in the cut stump treatment, mean biomass in manual removal plots was nearly as high and more than three times higher than in the basal bark treatment (18.84 ± 2.72 and 5.45 ± 0.89 cm, respectively). Restoration interventions and fencing had no significant overall effects on vegetation biomass (P > 0.05).

### Species richness and vegetation cover

**Fig. 5:**
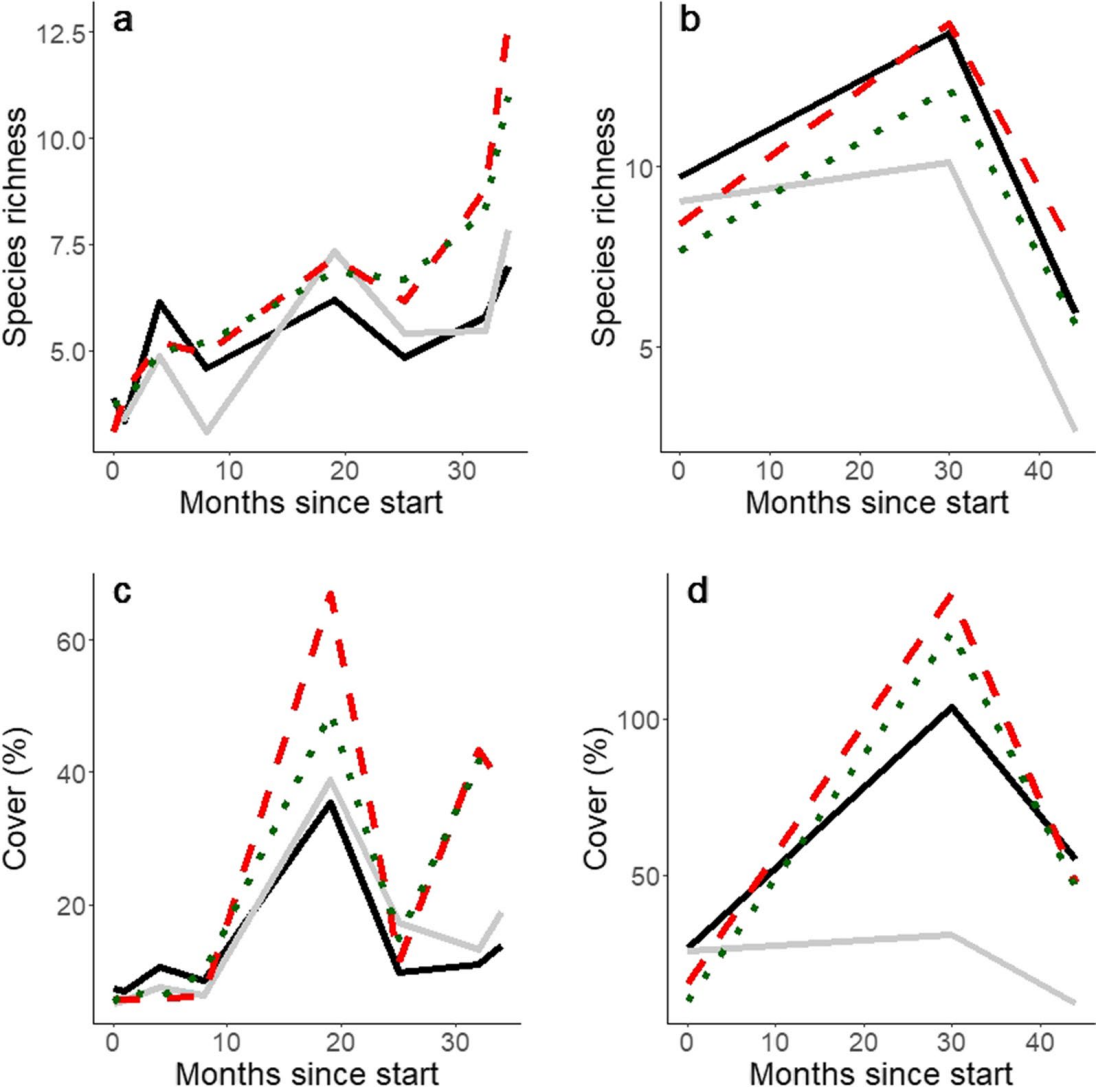
Mean plant species number and total estimated vegetation cover in prosopis treatments (top and bottom plots, respectively) at the Kenyan and Tanzanian sites over the course of the study (left and right plots, respectively). Solid grey, dotted green, dashed red and solid black lines indicate means for basal bark, cut stump, manual removal and control treatments

**Fig. 6:**
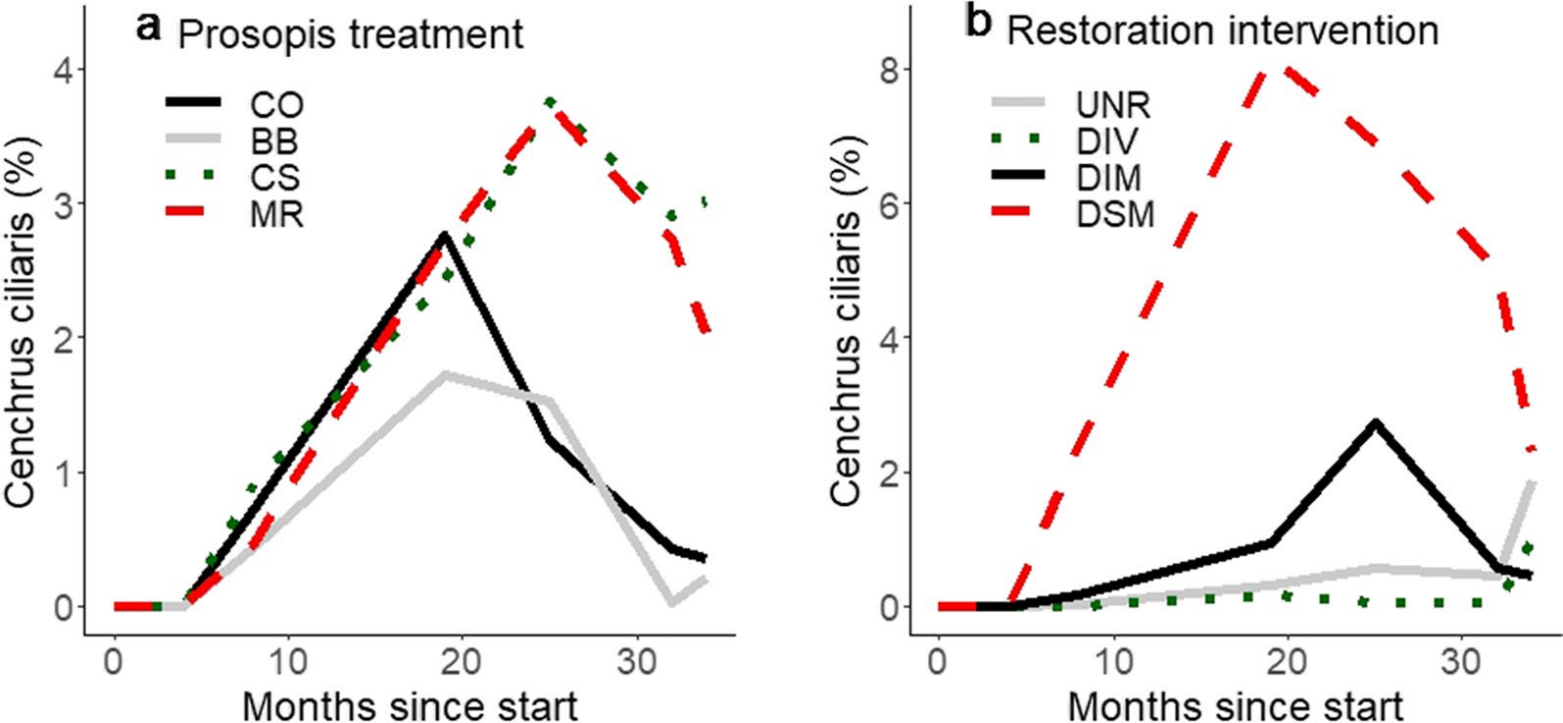
Abundance of *Cenchrus ciliaris* in Kenyan plots over the course of the study, separated by **a** prosopis management and **b** restoration treatments. In figure a, solid grey, dotted green, dashed red and solid black lines indicate means for basal bark, cut stump, manual removal and control treatments. In figure **b** the dotted green, solid black, dashed red and solid grey lines indicate divoting, divoting and mulching, divoting, mulching and sowing, and the unaided restoration treatments

Vegetation composition in Kenya and Tanzania was strongly affected by the prosopis treatments, and less so by restoration interventions. Species richness and cover were higher in treatments where aboveground prosopis biomass was removed, but this was more clearly seen in Kenya than in Tanzania (Fig. 5). In Tanzania species richness and cover were lower in control plots where no prosopis management was done than in the other treatments. The strongest effect of restoration interventions was seen where *Cenchrus ciliaris* was sown in Kenya, indicating that most vegetation established from seeds that were present in the seed bank, especially where prosopis was removed and the soil was disturbed by activities to implement the treatments (Fig. 6).

In Kenya, the mean number of plant species increased in all prosopis treatments over the course of the experiment (P < 0.001; Additional file 2; Fig. 5). However, the difference in total species richness among the prosopis treatments only became apparent from 25 months after the start of the experiment onwards and at the end of the study the number of species was ca. 1.5 times higher in cut stump and manual removal plots than in control and basal bark plots (prosopis treatment x months since start interaction: P < 0.001). No effects of the restoration interventions on species richness were found (P > 0.05; Additional file 2).

Total vegetation cover in Kenya was affected in a complex manner by all factors except for fencing (Fig. 5). Total vegetation cover varied over the course of the study (P < 0.001; Additional file 2), with an overall increase recorded, but the cover was higher eight months after the start of the experiment than during the recording period immediately prior and after it. Vegetation cover was ca. 2.5 times higher in cut stump and manual removal plots than in control and basal bark plots 25 months after the start of the experiment, but earlier in the study there were no differences among the prosopis treatments (prosopis treatment x months since start interaction: P < 0.001). This general pattern was observed in cover of perennial species, which was higher in manual removal and cut stump than in control and basal bark plots from eight months onwards. This was most clearly seen in the abundance of *Cenchrus ciliaris*, which became well established in all prosopis treatment plots during the first 18 months of the study and remained in the cut stump and manual removal, while its abundance declined in the control and basal bark treatments after month 19 (Fig. 6). While there were differences in vegetation cover among the restoration interventions (P < 0.001), and the restoration intervention effect varied across the prosopis treatments and across the study period (both P < 0.001), no clear patterns were found. However, perennial grass cover appears to have been higher in DSM than in UNR, DIV and DIM treatment plots for a number of the recording periods, which appears to have been caused by higher cover of *C. ciliaris*, the only species sown at this site, in DSM than in the other treatments from month eight to 32, as well as in CS and MR from month 25 onwards (Fig. 6), indicating that sowing was beneficial for the rapid establishment of that species.

Species richness in the Tanzanian plots varied over time and across prosopis treatments, with the number of species recorded 30 months after the start of the experiment almost two times higher than the number after 44 months, but the number of species in the control plots was lower than in the other prosopis treatments 33 and 40 months after the start of the experiment (prosopis treatment x months since start interaction: P < 0.001; Additional file 2; Fig. 5). Vegetation cover was, as in Kenya, affected by several experimental factors and their interactions. Like species richness, vegetation cover was highest 33 months after the start of the experiment and cover was lower in the control treatment than in the other prosopis treatments 33 and 40 months after the start (prosopis treatment x months since start interaction: P < 0.001). No clear effect of restoration interventions on vegetation cover was observed, although cover in DSM appeared to be lower than in the other interventions 33 months after start of the experiment. The large number of interactions among experimental factors and the lack of clear patterns appears to reflect variation among plots and blocks.

### Vegetation composition

**Fig. 7:**
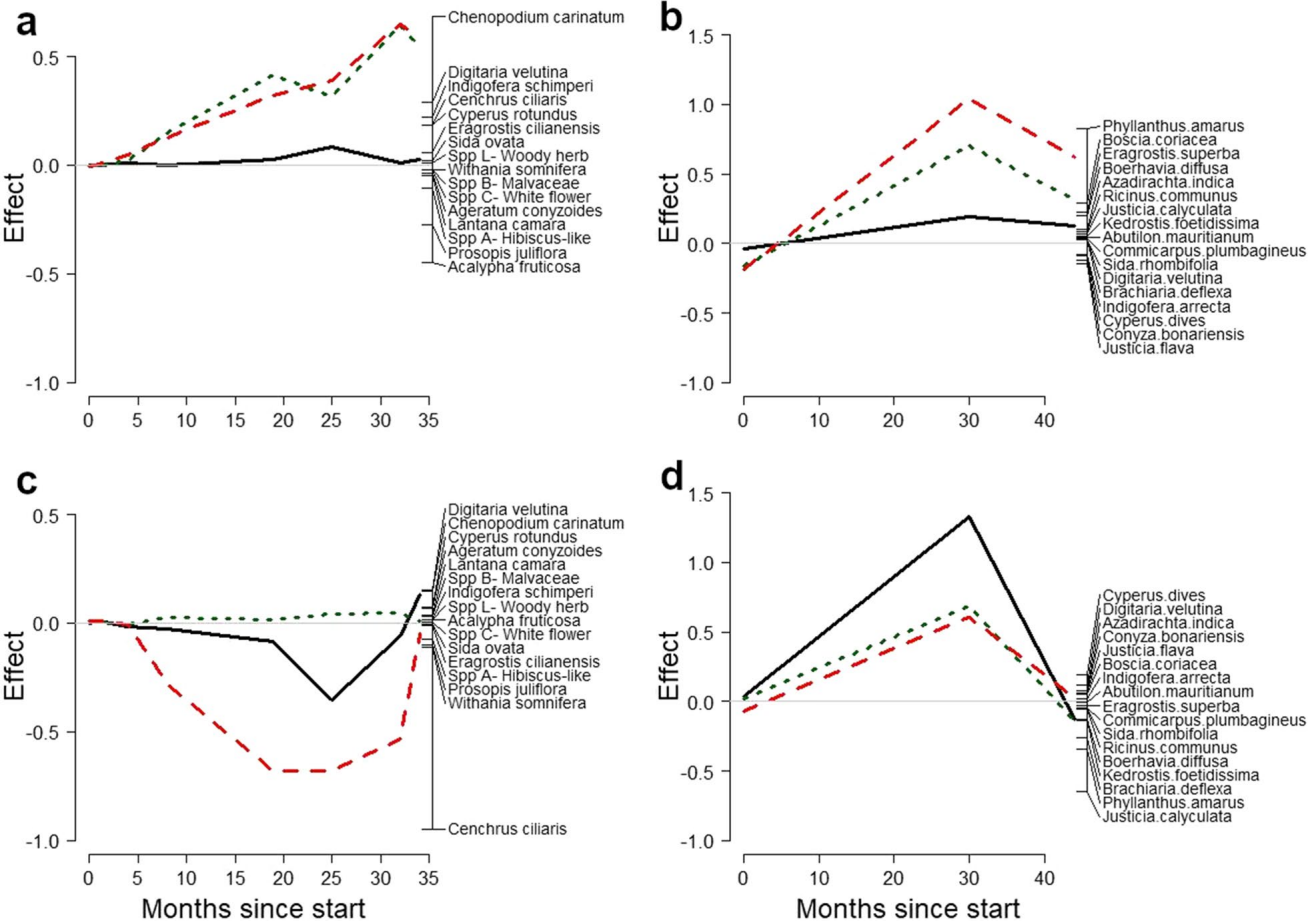
Principle Response Curves showing changes in vegetation composition over the course of the study as a result of prosopis treatments and restoration interventions in Kenya (**a** and **c**) and Tanzania (**b** and **d**). In each figure, the grey horizontal line through Y = 0 indicates the standardised control treatment, or unaided restoration treatment, and the other lines relative changes in species composition as a result of the treatments. In the top figures the dotted green, solid black, dashed red and solid grey lines indicate divoting, divoting and mulching, divoting, mulching and sowing treatments. In the bottom figures the dotted green, solid black, and dashed red lines indicate divoting, divoting and mulching, divoting, mulching and sowing treatments. Prosopis treatments explained 22.4% and 12.0% of the variation and restoration interventions 4.7% and 1.9% of the variation in vegetation composition in Kenya and Tanzania. Only species found on more than ten occasions (plots or plots x time) are shown

In Kenya, the vegetation in the cut stump and manual removal treatments became increasingly different from the vegetation in the control and basal bark treatments (PRC: F_24,736_ = 8.87, P = 0.001, 22.4% of variation explained; Fig. 7a), largely irrespective of the restoration interventions, which explained less of the variation (PRC: F_24,736_ = 1.51, P = 0.001, 4.7% of variation explained; Fig. 7c). This indicates that the effect of the prosopis treatments was larger than that of the restoration interventions. The change in vegetation composition appeared to be driven by a few species only. In the prosopis treatments, four species were associated with the control plots (*Acalypha fruticose* Forssk., *Prosopis juliflora*, *Solanum nigrum* L. and one unidenfied species (“Spp A- Hibiscus-like”)). *Lantana camara* L. and *Ageratum conyzoides* L. were associated with basal bark. Eight species were associated with cut stump [including four native perennial grasses: *Chenopodium carinatum* R. Br., *Cyperus rotundus* L., *Digitaria velutina* Forssk., *Eragrostis racemosa* (Thunb.) Steud., *E. tenuifolia* (A. Rich.) Hochst. ex Steud, *C. ciliaris*, *Ocimum basilicum* L., *E. ciliaris* (L.) R. Br., *Leucas martinicensis* (Jacq.) R.Br. and one unidentified species (“Sp5 new”)] and six with manual removal (four alien species and one annual grass: *Datura stramonium* L., *Indigofera schimperi* Jaub. & Spach, *E. cilianensis* (All.) Vignolo ex Janch., *Eragrostis* spA, *Bidens pilosa* L. and *Alternanthera pungens* Kunth). Only few plant species were significantly associated with any of the treatments (indicator species analysis: P < 0.05). *C. ciliaris* and *Withania somnifera* (L.) Dunal were significantly associated with DSM, although *C. ciliaris* was the only sown species. No species were associated with any of the other restoration interventions.

The prosopis treatments also had clear effects on vegetation composition in Tanzania, with manual removal and cut stump being the most different from the control treatment (PRC: F_9,276_ = 5.00, P < 0.001, 12.0% of variation explained; Fig. 7b). *Glycine wigthii* (Wight & Arn.) J.A. Lackey was associated with DSM and no other species were associated with restoration interventions (indicator species analysis: P < 0.05). Seven species were associated with basal bark (two alien species and five native herbs: *Conyza bonariensis* L., *Launaea cornuta* (Hochst. ex Oliv. & Hiern) C.Jeffrey, *Tamarix nilotica* (Ehrenb.) Bunge, *Indigofera arrecta* Hochst. ex A.Rich., *Kedrostis foetidissima* (Jacq.) Cogn., *Senna bicapsularis* (L.) Roxb. and *Melhania velutina* Forssk.), five with cut stump (both alien and native, including one native perennial grass: *Boerhavia diffusa* L., *C. rotundus*, *E. superba* Peyr., *Abutilon mauritianum* (Jacq.) Medik. and *Momordica dipsaceus* Ehrenb. ex Spach), and just three with manual removal: *Phyllanthus amarus* Schum. & Thonn., *Boscia coriacea* Pax and *Azadirachta indica* A.Juss. No species were associated with the control. *Phyllanthus amarus* was very dominant in the manual removal treatment 30 months after treatment implementation. The effect of restoration interventions on vegetation composition was not significant (Fig. 7d).

## Discussion

Our results illustrate how combined prosopis management and restoration interventions can result in grassland vegetation within few years, thus reversing some of the impacts of prosopis and providing livelihood support. Although our study was done in fairly small plots, such demonstrations are important, because these can support selection of the most cost-effective measures and show local actors that it is feasible to clear an area of prosopis and make the land suitable for other land uses, such as cattle grazing or the production of fodder.

### Effects and cost of prosopis treatment

The effectiveness of the treatments tested in the three countries was high and comparable, with the exception of the basal bark treatment in the Kenyan field trial. This may be the result of differences in stem base diameter among the three trial sites; the trees in Kenya were larger than those in Tanzania and Ethiopia (B. Megersa, pers. obs.). In Kenya, 16.6% of the measured stems had as stem base diameter of > 10 cm and 53.6% a diameter of > 5 cm, whereas in Tanzania less than 1% of the trees had a stem based diameter of > 10 cm and only 22.0% had a diameter of > 5 cm. Treatment effectiveness is known to be inversely related to the diameter of treated stems of woody species (e.g. Enloe et al. 2016; Kline and Duquesnel 1996; Worwood and Patterson 2011), which can be remediated by adapting the dose of Triclopyr to the stem diameter (e.g. Denny and Goodall 1992).

Our results largely support the findings from earlier studies and recommendations made for Australia and other regions. The fact that in Tanzania 22% of the stems had a stem base diameter of > 5 cm but basal bark treatment nevertheless reached an effectiveness of 91.3% suggests that this approach is also suitable for stems slightly larger than 5 cm stem base diameter. In Kenya, a significant number of stems was thicker than what is recommended for basal bark spraying, but the high effectiveness found for cut stump treatment (92.8%) supports earlier findings that the cut stump treatment has a broader range of application with regard to stem base diameter.

In Australia, both basal bark and cut stump treatments are recommended for prosopis management (New South Wales 2021). The choice between these two methods depends on a number of factors, including the diameter and the density of the trees, costs and the available manpower. Basal bark treatment is particularly suitable for use in scattered, low density and medium-density infestations, and also for regrowth of trees that have not been killed by a previous treatment application. According to Osmond et al. (2003), the effectiveness of basal bark spraying is greater than 97% mortality rate if carried out correctly. Cut stump is also suitable for prosopis management in scattered and low-density situations and may, if properly used, result in even higher kill rates and requires less chemicals than basal bark spraying (Osmond et al. 2003). The cut stump treatment, however, is time-consuming and considerably more labour intensive. Also, it is desirable to apply the cut stump treatment in two-person teams, one for cutting and the other for applying the chemical, adding to the cost. Moreover, our experience in the Tanzanian site reveals that removing the cut trees can be cumbersome if the branches in the canopy are intertwined, increasing the required time and cost.

Removal of prosopis through uprooting is labour-intensive and may only be needed if the subsequent land use involves ploughing. In other cases, cut stump may be the most appropriate method: it allows livestock access to the herbaceous vegetation, which is not or less the case if the stems are left in place following basal bark herbicide application. Moreover, the vegetation richness and composition data suggest that, contrary to our hypothesis, removal of aboveground prosopis biomass is beneficial for vegetation establishment. In addition, although our results show that the labour cost of cut stump is higher than the cost of basal bark, cut stump provides direct benefit if the removed wood is used for charcoal making that offsets some of the removal and restoration costs (Eschen et al. 2021a, b). Leaving aboveground biomass vs removal is not necessarily a question of cost, however, as basal bark can be labour intensive if the branches in the understory form a thicket that needs to be navigated.

### Vegetation development and restoration interventions

Sowing had a clear effect in Kenya, especially visible as the comparatively rapid and significant establishment of the grass *Cenchrus ciliaris*. The establishment of the species was most abundant and lasting in manual removal and cut stump treatments (reaching average 8–10% cover), suggesting that soil disturbance had a positive effect. This appears consistent with the findings of Visser et al. (2007), who found that a combination of tilling, seeding and application of brush cuttings yielded best establishment of sown species. The achieved cover of *C. ciliaris* in those treatments may not be considered very abundant, but the treatment didn’t aim to create vegetation dominated by a single species and the plant species that established in the experimental plots appear to have largely established from the soil seed bank and were quite numerous, indicating that the seed bank was still diverse, although there seems to have been quite some variability in composition (see next paragraph). The restoration treatments that were tested in this study were additive and it is therefore not possible to assess whether the establishment of *C. ciliaris* also benefited from the mulching. However, this species is commonly sown as seed, without mulching, for production of seed, fodder and hay for thatching in Baringo (Githu 2020; Lugusa et al. 2016), suggesting that seed addition alone is sufficient for good establishment of the species. Perennial grasses are important in the ecosystem because they stabilise the soil, sequester carbon and provide fodder, especially those that are resistant against grazing or trampling by animals, such as *C. ciliaris* (Mganga et al. 2015). The data on species richness and cover indicate that although *C. ciliaris* was the most abundant, perennial grasses established faster than some of the other species groups, illustrating that removal of prosopis can result in establishment of quality grassland.

There were large differences in vegetation composition between the sites, which is likely a reflection of differences in circumstances, such as site history and the soil seed bank. The sowing treatment facilitated establishment of grass species and thus increased the speed of succession, but no evidence was found for the addition of species as a result of hay addition, which may be the result of the use of dry hay, rather than freshly cut grass for the mulch. Many seeds are lost during hay making (Kiehl et al. 2010) and the main objective of the hay in our study was to create a microclimate beneficial for the germination and establishment of seedlings, but we found no evidence of the benefits of only adding hay on vegetation biomass or composition. Similarly, the results don’t provide support for benefits of creating topographic structures like divots that were intended to collect seeds, organic matter and rain water to support seedling establishment (Wagner et al. 2016). Other studies, often in different habitat types, have found that adding topographic structures can be successful in promoting vegetation establishment (Biederman and Whisenant 2011; Vivian-Smith 1997; Xie et al. 2019) and one should not generalize from the findings of our study.

The species associated with manual removal were generally weedy species, including known invasive species, whereas indicator species in the cut stump treatment were either grasses or herbaceous dicot species. This suggests that manual removal, perhaps as a result of the extensive soil disturbance, favoured establishment of ruderal species. The lower disturbance associated with cut stump treatment resulted in best establishment of grasses and herbaceous species. The indicator species associated with cut stump included several *Eragrostis* species, a genus that includes grasses preferred by pastoralists (Mganga et al. 2015).

The lower herbaceous biomass in the control and basal bark treatment plots and the (nonsignificant) difference in herbaceous biomass between these two treatments may be due to a number of factors, including shading, which was higher in the control plots than in the basal bark treatment due to the presence of foliage. Lower disturbance of the thick litter layer under prosopis stands might be expected to inhibit establishment of herbaceous vegetation, but we did not see a consistent positive effect of divoting in those treatments. Yet, some of the species established in the manual removal and cut stump treatments, including *Datura stramonium*, *Bidens pilosa* and *Alternanthera pungens*, are also light demanding and therefore cannot grow under shade, a factor that may also have promoted establishment of *C. ciliaris* and even of *P. juliflora* seedlings. Similarly, most prosopis seedlings were found in cut stump without mulching, suggesting that shading or the absence of soil disturbance represents suboptimal conditions for prosopis regeneration, whereas the disturbance during uprooting may have buried the seeds too deep for germination (El-Keblawy and Al-Rawai 2005; Morgan et al. 2017). The emergence of prosopis seedlings following the removal of prosopis highlights the need for follow-up weeding, for a period of at least two years (Eschen et al. 2021a, b).

Removal of one IAS may promote the establishment of other alien species (e.g. Clewley et al. 2012), but rapid establishment of vegetation cover through seeding may limit this risk (Gentili and Citterio 2021). Establishment of perennial herbaceous species may be preferred over annuals, as such species may be indicative of stable vegetation composition and natural vegetation succession tends to yield an increasing fraction of perennial grasses (Kelemen et al. 2017). In our study, however, the fraction perennial species did not consistently increase over time. While the cover of perennial grasses did increase and reached its highest cover at the end of the study in Kenya (ca. 7% 35 months after the start of the experiment), in Tanzania the highest cover (ca. 30%) was recorded 30 months after the start of the experiment and strongly declined afterwards, possibly due to the dominant occurrence of *Phyllanthus amarus* in month 30, which may have negatively affected other species through competition or allelopathic effects. Some of the established species were alien, but our results do not suggest that alien species were more important than native species, as the number of native species was higher than that of alien species.

Many grassland habitats in Eastern Africa are degraded as a result of overgrazing and other disturbances, which can facilitate prosopis invasion (Linders et al. 2019). Restoration of grassland, on small scale for production of hay and seed or on larger scales for use as pasture, therefore is most likely to be successful if the grazing intensity is adapted to allow establishment of the vegetation following prosopis removal, set seed and reseed for at least two years. This may be achieved on small plots by erecting and maintaining fencing, but on larger areas that are communal grazing land, where fencing is impractical or too costly to implement, traditional systems of regulating grazing under the authority of community elders, may be used (Mureithi et al. 2014; Wairore et al. 2015).

### Conclusion and recommendations

This study shows that it is possible to kill prosopis and establish productive grassland in its place and illustrates the context dependency of the best methods for managing woody IAS (Masters and Sheley 2001). The results demonstrate that the cost (i.e. time investment) and subsequent vegetation development differ among the method employed for killing prosopis trees and the choice for one method over another depend on a combination of available resources, sizes of the target trees, available manpower and the intended land use following prosopis management. Both chemical control treatments are far cheaper and quicker than uprooting, with basal bark treatment even quicker than cut stump treatment. It might therefore be reasonable to use basal bark and cut stump treatments primarily at the invasion front as part of an integrated prosopis management plan to remove satellite populations which may become the source of new invasions (Van Klinken and Campbell 2009). Most of the restoration practices did not have significant impact on the species re-establishment and further studies need to elucidate whether the initial investment in prosopis treatment is offset by (economic) benefits provided by grassland established following removal of prosopis. Yet, these results indicate that significant livelihood benefits may be obtained as a result of the increased availability of fodder (Eschen et al. 2021a, b).

### Supplementary Information

**Additional file 1:** Table indicating dates of treatment application and data recording in the three countries.

**Additional file 2:** ANOVA-style tables presenting results of the univariate analyses: treatment implementation, biomass and species richness.

## Data Availability

The data used in this manuscript are available at 10.34857/0017114.
