# Supplementary material for: Experimental prosopis management practices and grassland restoration in three Eastern African countries
Source: CABI Agric Biosci. 2023 Jul 13;4(1):21. doi: 10.1186/s43170-023-00163-5 (PMC11116197; doi:10.1186/s43170-023-00163-5)
Supplement: Supplementary file 1 — Additional file 1: Table indicating dates of treatment application and data recording in the three countries. [file 43170_2023_163_MOESM1_ESM.docx]

Supplementary Material to “Experimental prosopis management practices and grassland restoration in three Eastern African countries”

Additional File 3. Dates of treatment application and data recording in the three countries.

| Parameter assessed | Months since start | | |
| --- | --- | --- | --- |
|  | Ethiopia | Kenya | Tanzania |
| Stem-base diameter | NA | 0 | 0 |
| Prosopis treatment application | 0 | 0 | 0 |
| Implementation time | 0 | 0 | 0 |
| Tree survival | 3, 6, 9 | 33 | 7, 19 |
| Emerging seedlings | NA | 7, 8, 15, 20 | NA |
| Vegetation composition | NA | 0, 1, 4, 8, 19, 25, 32, 34 | 0, 30, 44 |
| Vegetation biomass | NA | NA | 44 |
